# CRISPR/Cas9-mediated TOP1 knockout in chicken DF-1 cells reveals its critical role in apoptosis regulation and genomic stability

**DOI:** 10.1093/jas/skaf315

**Published:** 2025-09-15

**Authors:** Xiaoqian Lv, Qiang Wei, Qiong Zhi, Xin Liu, Fan Li, Yingjie Niu, Hongyan Sun, Kai Jin, Guo hong Chen, Bichun Li, Qisheng Zuo

**Affiliations:** Joint International Research Laboratory of Agriculture and Agri-Product Safety of Ministry of Education of China, Yangzhou University, Yangzhou 225009, China; Key Laboratory of Animal Breeding Reproduction and Molecular Design for Jiangsu Province, College of Animal Science and Technology, Yangzhou University, Yangzhou 225009, China; Joint International Research Laboratory of Agriculture and Agri-Product Safety of Ministry of Education of China, Yangzhou University, Yangzhou 225009, China; Key Laboratory of Animal Breeding Reproduction and Molecular Design for Jiangsu Province, College of Animal Science and Technology, Yangzhou University, Yangzhou 225009, China; Joint International Research Laboratory of Agriculture and Agri-Product Safety of Ministry of Education of China, Yangzhou University, Yangzhou 225009, China; Key Laboratory of Animal Breeding Reproduction and Molecular Design for Jiangsu Province, College of Animal Science and Technology, Yangzhou University, Yangzhou 225009, China; Joint International Research Laboratory of Agriculture and Agri-Product Safety of Ministry of Education of China, Yangzhou University, Yangzhou 225009, China; Key Laboratory of Animal Breeding Reproduction and Molecular Design for Jiangsu Province, College of Animal Science and Technology, Yangzhou University, Yangzhou 225009, China; Joint International Research Laboratory of Agriculture and Agri-Product Safety of Ministry of Education of China, Yangzhou University, Yangzhou 225009, China; Key Laboratory of Animal Breeding Reproduction and Molecular Design for Jiangsu Province, College of Animal Science and Technology, Yangzhou University, Yangzhou 225009, China; Joint International Research Laboratory of Agriculture and Agri-Product Safety of Ministry of Education of China, Yangzhou University, Yangzhou 225009, China; Key Laboratory of Animal Breeding Reproduction and Molecular Design for Jiangsu Province, College of Animal Science and Technology, Yangzhou University, Yangzhou 225009, China; Joint International Research Laboratory of Agriculture and Agri-Product Safety of Ministry of Education of China, Yangzhou University, Yangzhou 225009, China; Key Laboratory of Animal Breeding Reproduction and Molecular Design for Jiangsu Province, College of Animal Science and Technology, Yangzhou University, Yangzhou 225009, China; Joint International Research Laboratory of Agriculture and Agri-Product Safety of Ministry of Education of China, Yangzhou University, Yangzhou 225009, China; Key Laboratory of Animal Breeding Reproduction and Molecular Design for Jiangsu Province, College of Animal Science and Technology, Yangzhou University, Yangzhou 225009, China; Joint International Research Laboratory of Agriculture and Agri-Product Safety of Ministry of Education of China, Yangzhou University, Yangzhou 225009, China; Key Laboratory of Animal Breeding Reproduction and Molecular Design for Jiangsu Province, College of Animal Science and Technology, Yangzhou University, Yangzhou 225009, China; Department of Food technology, College of Biochemical Engineering, Yangzhou Polytechnic College, Yangzhou, China; Joint International Research Laboratory of Agriculture and Agri-Product Safety of Ministry of Education of China, Yangzhou University, Yangzhou 225009, China; Key Laboratory of Animal Breeding Reproduction and Molecular Design for Jiangsu Province, College of Animal Science and Technology, Yangzhou University, Yangzhou 225009, China; Department of Food technology, College of Biochemical Engineering, Yangzhou Polytechnic College, Yangzhou, China; College of Biotechnology, Jiangsu University of Science and Technology, Zhenjiang 212100, China; Joint International Research Laboratory of Agriculture and Agri-Product Safety of Ministry of Education of China, Yangzhou University, Yangzhou 225009, China; Key Laboratory of Animal Breeding Reproduction and Molecular Design for Jiangsu Province, College of Animal Science and Technology, Yangzhou University, Yangzhou 225009, China

**Keywords:** apoptosis, clustered regularly interspaced short palindromic repeats/CRISPR-associated protein 9, DF-1 cells, genomic stability, topoisomerase I

## Abstract

The role of topoisomerase I (encoded by TOP1) in avian cell survival and apoptosis regulation remains unclear, limiting its potential application in poultry biotechnology. This study aimed to establish a CRISPR/Cas9-mediated TOP1 knockout platform in chicken DF-1 cells and evaluate its functional impact on apoptosis. Three sgRNAs targeting TOP1 were designed and delivered via liposome vectors, achieving knockout efficiencies up to 50% as confirmed by T7 Endonuclease I (T7E1) assay and Sanger sequencing, with no detectable off-target effects. Functional analysis revealed that TOP1 knockout significantly increased apoptosis rates and upregulated DNA damage markers (γH2AX) and apoptotic genes (Caspase 8 and BRCA1). These results demonstrate that TOP1 is essential for maintaining genomic stability in avian somatic cells, and its depletion triggers apoptosis through DNA damage accumulation. Although synthetic lethality-based sex control was not directly tested here, our findings provide critical evidence that TOP1 dysfunction could theoretically enable selective elimination of specific cell populations (e.g., primary germ cells) via CRISPR editing. Notably, developing targeted delivery systems for PGCs—a focus of future research not addressed in this study—will be essential to achieve such selectivity in vivo, highlighting a significant technological hurdle to overcome.

## Teaser

By removing a key DNA-stabilizing enzyme (TOP1) in chicken cells using CRISPR gene editing, researchers confirmed its vital role in preventing cell death and DNA damage—a breakthrough that could pave the way for ethical strategies to manage poultry sex ratios.

## Introduction

The importance of sex control in poultry production cannot be overstated, as the poultry industry is a vital component of the global agricultural economy, with production efficiency closely tied to sex ratio control (Zhang et al., 2023). In egg-laying chicken production, hens exhibit far greater productivity than roosters, while in broiler production, roosters often surpass hens in growth rate and meat quality (Mueller et al., 2023). Consequently, achieving single-sex production in poultry is crucial for enhancing both production efficiency and economic benefits within the poultry industry (Timothy et al., 2016). However, due to the incomplete understanding of the sex determination mechanism in chickens, traditional methods of sex identification and culling not only increase production costs but also result in significant resource wastage and raise ethical concerns (Timothy et al., 2016). Therefore, the development of novel sex control technologies to reduce chick culling rates has become an urgent issue in the poultry breeding industry.

The rapid advancement of Clustered Regularly Interspaced Short Palindromic Repeats/CRISPR-associated protein 9 (CRISPR/Cas9) technology has enabled direct manipulation of sex-determining genes (Cong and Zhang, 2015; Timothy et al., 2016; Jason et al., 2021). This breakthrough has presented unprecedented opportunities for the poultry industry to implement more precise sex control strategies and sparked scientific inspiration to explore more diverse pathways for sex regulation. The removal of the specific Y-chromosome gene Rbmy1a1 in male mouse embryonic stem cells using CRISPR-Cas9 technology successfully eliminated the Y-chromosome, resulting in pluripotent XO chromosome stem cells and the cultivation of fertile mice with a phenotypic sex transition from male to female (Qin et al., 2021). Knockout of the HMG domain of the Sex-determining Region Y gene (SRY) in pigs led to sex reversal in gene-edited pigs (Kurtz et al., 2021). In poultry, while CRISPR-Cas9 technology has been used to knockout genes related to sex control, true sex determination control has yet to be achieved (Jason et al., 2021).

The development of efficient poultry sex—control technology holds great significance for reducing chick culling rates, enhancing production efficiency and economic returns in the poultry industry, promoting rational resource utilization, and alleviating the burden on animal welfare, thus contributing to the sustainable development of the poultry farming sector. Breakthroughs in biotechnology, particularly the successful application of the “synthetic lethality” strategy for mouse sex control, have unlocked new possibilities for innovating poultry sex control technology (Wu et al., 2019; Douglas et al., 2021). The TOP1 gene, a critical factor in maintaining DNA stability and regulating the cell cycle (Berroyer and Kim, 2020; Keith, 2022), serves as a potential target for sex control due to its dysfunction triggering DNA damage and cellular apoptosis (Sordet et al., 2003; Kaja et al., 2018). In this study, we precisely edited the TOP1 gene using CRISPR/Cas9 technology and identified suitable sgRNA anchoring the TOP1 gene (TOP1-sgRNA), laying the foundation for exploring the integration of sgTOP1 or Cas9 into safe sites on the Z or W chromosomes (Wu et al., 2024). This represents a significant step toward developing poultry sex-control technology. Specifically, our strategy for generating single-sex chicken models in subsequent research involves knocking TOP1-sgRNA and Cas9 fragments into the safe sites of the Z chromosome (previously identified by our team) in ZZ-type PGCs to generate F1 chimeric chickens. When their gametes combine to produce F2 gene-edited chickens, selecting Z^cas9^W and Z^TOP1-sgRNA^Z^TOP1-sgRNA^ gene-edited chickens for mating will exclusively yield Z^TOP1-sgRNA^W hens, thus achieving single-sex chicken model construction.

## Materials and Methods

### Ethical statement

The animal experiments were approved by the Institutional Animal Care and Use Committee of the Animal Experiment Ethics Committee of Yangzhou University (License Number: SYXK [Su] IACUC 2012-0029). All experimental procedures were conducted in accordance with the “Regulations for the Administration of Laboratory Animals” approved by the State Council of the People’s Republic of China.

### Sequence, homologous, and phylogenetic analysis of the TOP1 gene of *Gallus gallus* (*Gg*TOP1)

To characterize the structural and evolutionary features of the chicken TOP1 gene and its protein product, we conducted multi-level analyses. A translation tool (http://web.expasy.org/translate/) was used to predict the amino acid sequence of *Gg*TOP1. SMART (http://smart.embl-heidelberg.de/) was utilized to predict the domain of *Gg*TOP1. Amino acid sequences of TOP1 from different species were collected from the NCBI GenBank (http://www.ncbi.nlm.nih.gov/genbank/) for sequence alignment using online sequence analysis (http://www.bio-soft.net/sms/index.html). A phylogenetic tree of TOP1 was constructed with MEGA 10.0.

### Construction of vector

To enable precise CRISPR/Cas9-mediated editing of the *GgTOP1* gene, we designed and constructed sgRNA-expression vectors. This step was critical for delivering gene-editing components to target cells and achieving site-specific DNA cleavage. From the NCBI database, the exon (coding) regions of the TOP1 gene were downloaded. The CRISPOR online website (http://crispor.tefor.net/) was employed to predict sgRNAs for the exon (coding) regions of exons 4, 13, and 12. sgRNAs with high-scoring rates and low-mismatch rates were selected as candidate sgRNAs. A total of three corresponding sgRNAs were designed, namely sgRNA1, sgRNA2, and sgRNA3. The target sequences of the three sgRNA target sites could be found in Table 1. Oligo primers of TOP1-sgRNA1, TOP1-sgRNA2, and TOP1-sgRNA3 were synthesized according to the enzyme digestion base sequences (Supplementary Table S1) and annealed to form double-stranded DNA, which was then linked to the vector PX458 after BbSI digestion (NEB, USA) with T4 ligase (Takara, Dalian, China). The PX458 vector (Addgene #48138; Beijing Tsingke Biotech Co., Ltd) was used for CRISPR-Cas9-mediated gene editing. This vector expresses Streptococcus pyogenes Cas9 fused to an EGFP tag at the C-terminus. Following the instructions of the Trelief 5α Chemically Competent Cell (Tsingke, Beijing, China), the mix products of the vector and the Trelief 5α Chemically Competent Cell were incubated on ice for 5 min, heat-shocked at 42 °C for 45 s, chilled on ice for 2 min, plated on solid culture medium containing ampicillin, and incubated at 37 °C for 16 h. Single colonies were picked for further Sanger sequencing. The correctly sequenced mono-clones were placed into LB medium for amplification culture and plasmid extraction. Plasmid extraction was performed using the EndoFree Maxi Plasmid Kit (Tiangen, Beijing, China) according to the manufacturer’s instructions.

**Table 1.** Targeted knockout of TOP1 gene sgRNA sequences

Gene	The sequence of sgRNA primers(5′→3′)
**TOP1-sgRNA1**	F: CAAAGACAAGGAGAAGAGAAAGG
	R: GTTTCTGTTCCTCTTCTCTTTCC
**TOP1-sgRNA2**	F: GGAATATGGTTACTGTGTGATGG
	R: CCTTATACCAATGACACACTACC
**TOP1-sgRNA3**	F: GGAGGTCCGGCATGATAACAAGG
	R: CCTCCAGGCCGTACTATTGTTCC

### Cell culture

To establish a suitable system for CRISPR/Cas9 transfection and gene-editing validation, chicken fibroblast cells DF-1 (maintained in our laboratory) were used for in vitro culture. This step provided a controlled environment to assess vector efficiency and cellular responses to TOP1 knockout. DF-1 (preserved in our laboratory) was cultured in Dulbecco’s Modified Eagle Medium (DMEM) medium (Gibco, USA) containing 10% HyClone Defined Fetal Bovine Serum (FBS) (Cytiva, USA) and 1% Penicillin-Streptomycin Solution (P/S). DF-1 cells were adjusted to a density of 1 × 10^5^ cells/mL and cultured until reaching 80% confluency. According to the instructions of the Lipo8000 Transfection Reagent kit (Beyotime, China), 1 μg of plasmid and 1.5 μL of transfection reagent were mixed in 25 μL of Opti-MEM I Reduced Serum Medium (Thermo Fisher Scientific, USA). The mixture was added to the cells, which were cultured in a total volume of 300 μL per well. The remaining volume was supplemented with DMEM containing 10% FBS without 1% P/S. Fluorescence observation and sample collection were conducted 48 h post-transfection.

### Screening of positive cells and RNA extraction

To isolate cells successfully transfected with the CRISPR/Cas9 vector and ensure accurate downstream gene-expression analysis, we employed fluorescence-activated cell sorting (FACS) and rigorous RNA extraction. The positive cells were screened via fluorescence-activated cell sorting (FACS) using a FACS Aria SORP flow cytometer. Since our vector, based on the PX458 backbone, carried an EGFP tag, we detected green fluorescence signals to sort and collect the transfected DF-1 cells for RNA extraction. The remaining sorted cells were kept in culture for further analysis. Total RNA from each group was extracted using TRIzol reagent (Tiangen, China). RNase-free DNase I (Takara, Dalian, China) was added to the reaction mixture for 10 min to effectively remove genomic DNA. The purity and concentration of RNA were measured using a NanoDrop 1000 spectrophotometer (Thermo Scientific, USA). The integrity of RNA was assessed using an Agilent 2100 Bioanalyzer (Agilent Technologies, Santa Clara, CA, USA). The extracted RNA was then reverse transcribed, with three replicates for each group.

### Quantitative real-time PCR (qRT-PCR)

To assess the impact of TOP1 knockout on gene expression related to cell death, DNA repair, and damage, we performed qRT-PCR on sorted positive cells. RNA was reverse transcribed into cDNA with HiScript II Q RT SuperMix for qPCR (+gDNA wiper) reagent (Vazyme, Nanjing, China, R223-01). qRT-PCR was performed using the FastKing One-Step RT-PCR Kit with SYBR green (QIAGEN, Beijing, China, KR123). The mRNA levels of relevant genes were detected using the CFX-Connect Real-Time PCR Detection System (BIO-RAD, California, USA, 7500fast). The primer sequences for qRT-PCR are listed in Table 1. The qRT-PCR cycling conditions were as follows: initial denaturation at 95 °C for 30 s, followed by 40 cycles of 95 °C for 10 s and 60 °C for 30 s. The results were quantified using the 2^-ΔΔCt^ method to normalize relative to β-Actin (Livak and Schmittgen, 2001; Schmittgen and Livak, 2008). The genes examined included apoptotic-related genes (Caspase 8, BRCA1), homologous recombination repair genes (RAD51), and DNA damage markers (γH2AX), with β-actin serving as the internal reference gene. The sequence of qRT-PCR primers is shown in Supplementary Table S2.

### T7 endonuclease I (T7E1) assay

To quantify the gene-editing efficiency of CRISPR/Cas9 at the *Gg*TOP1 locus, we employed the T7 Endonuclease I (T7E1) assay, which detects mismatches in heteroduplex DNA formed between wild-type and mutated alleles. Genomic DNA was extracted from the transfected cells at a density of 1 × 10^6^ cells/mL using the TIANamp Genomic DNA Kit (Tiangen, China). According to the instructions of the PrimeSTAR Max DNA Polymerase kit (TaKaRa, Dalian, China), the extracted DNA served as a template for PCR amplification. Primers were designed to amplify a region spanning approximately 200–250 bp upstream and downstream of the sgRNA target site (about 200–250 bp upstream and 200–250 bp downstream of the sgRNA locus) to capture potential indels induced by CRISPR/Cas9. The amplification used T7E1-specific primers (Supplementary Table S3), TOP1-sgRNA1 and TOP1-sgRNA2, and the enzyme digestion system and grouping are detailed in Supplementary Table S4. The PCR products were then subjected to T7 Endonuclease I digestion according to the instructions of the T7 Endonuclease I kit (NEB, Ipswich, MA). The reaction conditions included initial denaturation at 95 °C for 10 min, followed by a touchdown PCR program: starting from 95 °C for 2 s and decreasing by 0.1 °C every 2 s until 75 °C, then decreasing by 0.1 °C every second until 16 °C, followed by incubation at 16 °C for 2 min, and finally, digestion at 37 °C for 30 min. Digestion products were analyzed by 1% agarose gel electrophoresis. The ImageJ software was used to analyze the gray value of the gel results. Knockout efficiency = gray value of mutant bands/total gray value of mutant and unmutated bands ×100%

### Original TA cloning assay

To precisely characterize CRISPR/Cas9-induced mutations at the *Gg*TOP1 locus, including single-base changes, we performed TA cloning followed by Sanger sequencing. Following the instructions of the 5 min TA/Blunt-Zero Cloning Kit (Vazyme, Nanjing, China), DNA fragments of approximately 500 bp before and after the knockout region were amplified by PCR and ligated into the T vector. The amplification primers were located in Supplementary Table S3 (TA clone/T7E1 enzyme digestion amplification primer sequences). The ligated products were then transformed into the Trelief 5α Chemically Competent Cell (Tsingke, Beijing, China). At least 15 individual colonies were picked from each LB agar plate and subjected to sequencing analysis.

### Hoechst 33342 staining

To evaluate more intuitively the effect of targeted knockout of the TOP1 gene on apoptosis in DF-1 cells, this study employed Hoechst 33342 staining followed by cell counting to observe the changes in apoptosis post-transfection. Hoechst 33342 is a fluorescent labeling reagent specific for live cells, wherein normal cells exhibit brightly stained blue fluorescent nuclei, while apoptotic cells display condensed and intensely stained nuclei. Employing the PX458 vector transfection as the control group, the TOP1-sgRNA1-PX458 vector with the highest knockout efficiency was selected as the experimental group. Live cell counts were conducted and statistically analyzed at 48, 60, and 72 h post-transfection, with comparisons made to the control group. For staining, an appropriate amount of Hoechst 33342 live cell staining solution (100X) (C1029, Beyotime, China) was evenly added to the culture medium to achieve a final concentration of 1X. The mixture was incubated with samples at 37 °C for 10 min. Subsequently, the staining solution was aspirated, and the cells were washed three times with PBS before observation under a fluorescence microscope (Leica, Weztlar, Germany, DMC6200). Statistical analysis was performed based on three repeated experiments.

### Analysis of apoptosis

Given the limitations of using the PX458 vector (expressing EGFP) for apoptosis assays, which rely on Annexin V-FITC and PI staining, we subcloned sgRNA1 and sgRNA2 into the PX459 vector backbone (Addgene #62988; Beijing Tsingke Biotech Co., Ltd). This vector contains a puromycin resistance cassette (puro), enabling antibiotic selection of transfected cells. Following transfection with Lipo8000 (Beyotime, China), cells were cultured in DMEM medium for 24 h before being subjected to puromycin selection at a concentration of 2 μg/mL for two consecutive rounds (each round lasting 48 h). Surviving colonies were pooled and expanded for subsequent apoptosis assays. The apoptosis ratio was analyzed using the Annexin V-FITC Apoptosis Detection Kit (Beyotime, China) to assess whether TOP1 knockout induces apoptosis in transfected cells. At 72 h after transfection, cells were harvested and resuspended in binding buffer containing Annexin V-FITC and PI according to the manufacturer’s instructions. The samples were analyzed by flow cytometry (FACScan; BD Biosciences, USA). Cells were discriminated into viable cells, necrotic cells, and apoptotic cells by using Flowjo 10.8.1 software, and then the percentages of apoptotic cells from each group were compared. Tests were repeated in triplicate.

### Off-target site prediction

To investigate potential off-target effects of TOP1-sgRNA1-PX458 and TOP1-sgRNA2-PX458, this study employed the CCTop-CRISPR/Cas9 target online predictor (https://cctop.cos.uni-heidelberg.de/index.html) to predict off-target sites for the three sgRNAs. Primer sequences for these off-target sites are listed in Supplementary Table S5. T7E1 and TA cloning assays were performed according to the methods described above to analyze potential cleavage at predicted off-target loci.

### Statistical analysis

To assess the significance of gene expression changes and apoptosis rates following TOP1 knockout, we performed statistical analyses on data from flow cytometry experiments, gene editing efficiency, off-target effect, qRT-PCR, and apoptosis experiments. Flow cytometry transfection efficiencies for TOP1-sgRNA1-PX458, TOP1-sgRNA2-PX458, and TOP1-sgRNA3-PX458 were compared using one-way ANOVA with Tukey’s post hoc test to identify significant differences between groups. In gene editing efficiency and off-target effect assay: for T7E1 Assay, Cleavage efficiencies were calculated using the above-mentioned formula and the data were analyzed using ImageJ software (NIH) and reported as mean ± SEM (*n* = 3 independent transfections); for TA Cloning assay, Knockout efficiency was determined by counting the number of colonies with edited bases (insertions/deletions) in sequenced colonies. For qRT-PCR Data, relative gene expression levels (calculated using the 2^−ΔΔCt^ method) were compared between sgRNA-treated groups (sgRNA1, sgRNA2) and the PX458 group. Each experiment was repeated in triplicate (*n* = 3 biological replicates), and results were expressed as mean ± standard error (SEM). Apoptosis rates (the percentages of the total, early, and late apoptotic cells) were compared between sgRNA-treated groups (sgRNA1, sgRNA2) and PX459 control groups using Student’s *t*-test. In the Hoechst 33342 staining assay, viable cell counts were normalized to the PX458 control group at each time point (48, 60, and 72 h). Data were analyzed using two-way ANOVA with Tukey’s post hoc test (GraphPad Prism 9.5.1). SPSS (IBM Statistical Package for the Social Sciences) was used to perform Student’s *t*-tests to compare mean values between two groups. Significance was defined as *P *< 0.05 (*) or *P *< 0.01 (**). GraphPad Prism 9.5.1 was used for generating graphs (e.g., bar charts for qRT-PCR data, scatter plots for flow cytometry) and for visualizing statistical comparisons.

## Results

### High conservation of *TOP1* across different species

Various studies have shown that the *TOP1* gene in mammals is capable of mediating cellular apoptosis (Sordet et al., 2003; Kaja et al., 2018). To predict the function of the *TOP1* gene in *Gallus gallus* (*Gg*TOP1), the conservation of TOP1 during species evolution was compared. Bioinformatics analysis revealed that the full-length cDNA of *Gg*TOP1 is 3765 bp, with an open reading frame of 2300 bp, encoding a polypeptide of 766 amino acid residues. The amino acid sequence of the TOP1 is highly conserved across different species, with similarities to the *Taeniopygia guttata* and *Anas platyrhynchos* exceeding 90%, and conservation with other species ranging between 60 and 80% (Supplementary Figure S1A). The predicted amino acid sequence of *Gg*TOP1 shares 91.16% homology with TOP1 of *Mus musculus* (*Ms*TOP1) (Supplementary Figure S1B). *Gg*TOP1 contains regions of low complexity, two coiled-coil domains, and a TOPEUc domain, similar to *Ms*TOP1 (Supplementary Figure S1C). The three-dimensional structures of *Gg*TOP1 and *Ms*TOP1 are similar, both exhibiting comparable α-helices and β-sheets (Supplementary Figure S1D). All these findings indicate that TOP1 is highly conserved across different species, leading to a preliminary hypothesis that *Gg*TOP1 may also regulate cellular apoptosis.

### Construction of targeted *TOP1* gene knockout vector and evaluation of vector activity

**Figure 1.**
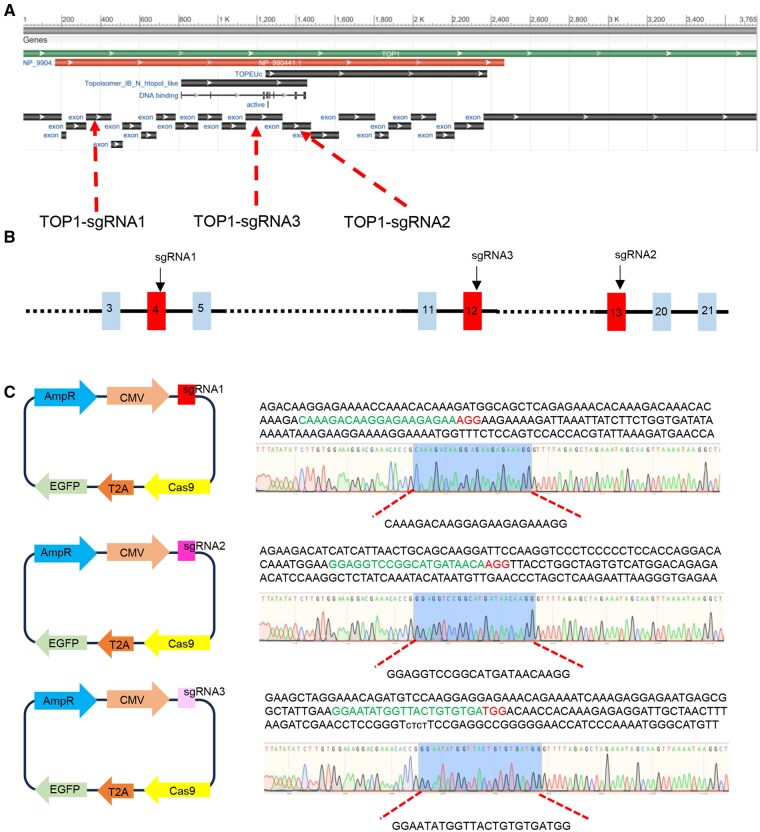
Construction of three vectors targeting TOP1. (A) Exon regions of the chicken TOP1 gene as predicted by NCBI. Red arrows indicate the locations of the three sgRNA target sites: TOP1-sgRNA1, TOP1-sgRNA2, and TOP1-sgRNA3. (B) Schematic of the design for the target sites of the three sgRNAs against TOP1. The numbers represent the exon positions, and the black boxes highlight the specific regions targeted by sgRNA1, sgRNA2, and sgRNA3. (C) Schematic illustration of the construction of TOP1-sgRNA-PX458 vectors. Each vector contains AmpR (ampicillin resistance gene), CMV (cytomegalovirus promoter), sgRNA (single-guide RNA), EGFP (enhanced green fluorescent protein) linked to Cas9 via T2A. The sequencing results below each vector schematic show the alignment of the sgRNA target regions. Red dashed arrows point to the key sequences in the alignment, highlighting the targeted nucleotide sequences for each sgRNA.

**Figure 2.**
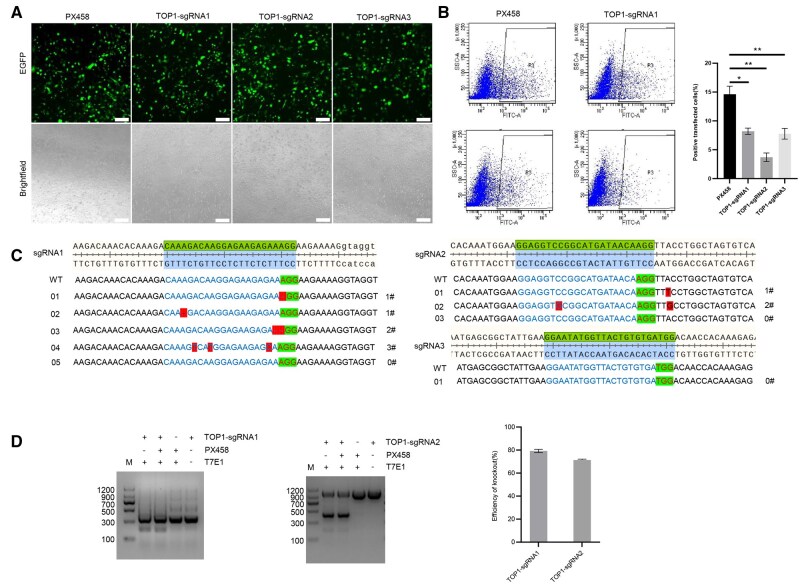
Detection of knockout efficiency after transfection of DF-1 cells with three knockout vectors targeting TOP1. (A) Fluorescence observation of DF-1 cells post-transfection with TOP1-sgRNA-PX458 knockout vectors. The top row shows EGFP fluorescence, and the bottom row shows brightfield images. Bar = 100 μm. PX458 indicates DF-1 cells transfected with the PX458 vector; Top1-sgRNA1, Top1-sgRNA2, and Top1-sgRNA3 represent DF-1 cells transfected with Top1-sgRNA1-PX458, Top1-sgRNA2-PX458, and Top1-sgRNA3-PX458 vectors, respectively. (B) Flow cytometry sorting of positive cells after transfection of DF-1 cells with TOP1-sgRNA-PX458 knockout vectors. The left panels display flow cytometry plots, and the right panel shows the statistical analysis of the percentage of positive cells. Asterisks denote statistical significance: *P *< 0.05 (*), *P *< 0.01 (**). (C) TA cloning assay of the flow cytometry-sorted positive cells. sgRNA1, sgRNA2, and sgRNA3 correspond to samples from DF-1 cells transfected with TOP1-sgRNA1-PX458, TOP1-sgRNA2-PX458, and TOP1-sgRNA3-PX458 vectors, respectively, followed by TA cloning assay. WT represents the sequencing result of the wild-type knockout target site. In TOP1-sgRNA1-px458 group: 0# indicates the type with no base deletion, 1# indicates the type with one base deletion, 2# indicates the type with two base deletions, and 3# indicates the type with three bases substitution. In TOP1-sgRNA2-px458 group: 0# indicates the type with no base deletion, 1# indicates the type with one base substitution, 2# indicates the type with two bases substitution. Red boxes represent PAM sequences. (D) T7E1 endonuclease assay of the sorted positive cells in the TOP1-sgRNA1-PX458 and TOP1-sgRNA2-PX458 groups. M represents the marker. Lanes are labeled as Test group, Hybrid group, Control group, and blank group. The right panel shows the quantification of the cleavage efficiency.

To specifically investigate the function of *Gg*TOP1, this study selected three exon regions for sgRNA targeting based on the domain analysis of the TOP1 gene (Figure 1A). Knockout vectors targeting exons 4, 13, and 12 of the TOP1 gene were constructed (Figure 1B), namely TOP1-sgRNA1-PX458, TOP1-sgRNA2-PX458, and TOP1-sgRNA3-PX458 (Figure 1C). The constructed knockout vectors were transfected into DF-1 cells, and positive cells were screened by flow cytometry 48 h post-transfection, yielding percentages of 14.63 ± 1.39%, 8.20 ± 0.55%, 3.7 ± 0.74%, and 7.77 ± 0.93%, respectively ­(Figure 2A and B). The control vector (PX458 alone) exhibited a higher transfection efficiency, with positive cell percentages significantly greater than those of the sgRNA-expressing vectors (Figure 2A and B). The results of the TA cloning sequencing experiment showed that the knockout efficiencies of TOP1-sgRNA1-PX458 and TOP1-sgRNA2-PX458 were 50% (5/10) and 20% (2/10), respectively (Figure 2C). The sorted positive cells were subjected to a T7E1 endonuclease assay. Clear cleavage bands were observed around the 200 bp position for TOP1-sgRNA1-PX458 and around the 400 bp position for TOP1-sgRNA2-PX458, indicating that all three sgRNA knockout targets possessed cleavage activity (Figure 2D). The cleavage efficiencies were 79.26 ± 4.95% and 71.39 ± 1.92%, respectively. Consistent with the T7E1 endonuclease assay results, the sequence at the TOP1-sgRNA1-PX458 site exhibited the highest knockout efficiency.

### Prediction of off-target sites and analysis of off-target effects of *TOP1* gene knockout vectors

**Figure 3.**
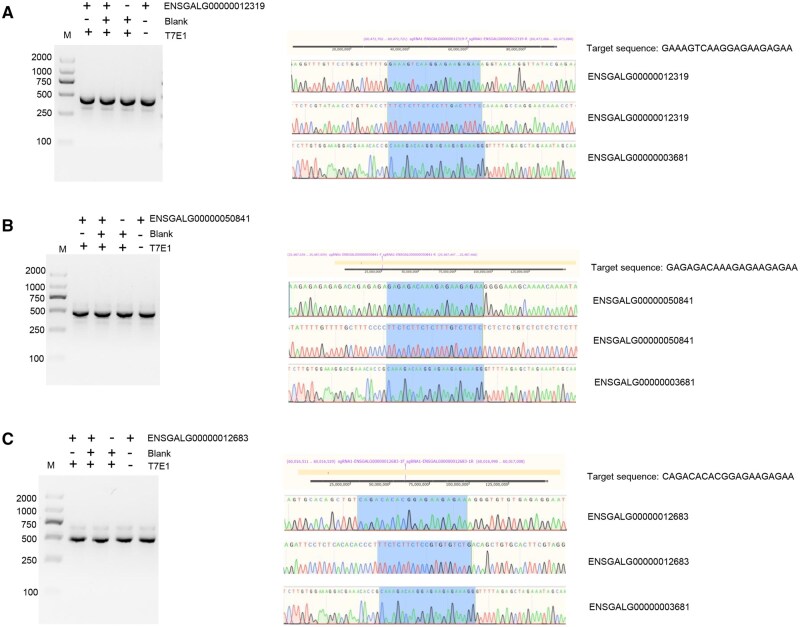
Detection of off-target probabilities for predicted off-target sites of TOP1-sgRNA1. (A–C) T7E1 endonuclease and TA cloning assays for genes with IDs ENSGALG00000012319, ENSGALG00000050841, and ENSGALG00000012683, respectively. In agarose gel plots, M represents the marker. TA cloning sequencing results show target sequences (blue boxes highlighted target sequences), with gene IDs indicated on the right. The lighter bands in the agarose gel plot were nonspecific amplification products. The lighter bands in the agarose gel plot were nonspecific amplification products.

To evaluate the specificity of the three TOP1-sgRNA-PX458 vectors, we focused on off-target sites with the highest predicted similarity to the sgRNA sequences, prioritizing coding regions (exons) and introns (Supplementary Figure S2). T7E1 endonuclease assays at these sites showed no specific cleavage bands (Figure 3; Supplementary Figure S3), and TA cloning of nine predicted off-target loci yielded amplified products that aligned with wild-type sequences in sequencing analyses, with no detected base deletions or mutations (Figure 3; Supplementary Figure S3). These results suggest no off-target effects at the evaluated sites. Notably, while our approach targeted high-probability off-target regions to balance comprehensiveness and specificity, we recognize that this does not constitute a genome-wide evaluation. Off-target prediction and validation in CRISPR studies remain inherently challenging, and our conclusions are limited to the sites tested herein.

### The effect of targeted knockout of *TOP1* gene on apoptosis in DF-1 cells

**Figure 4.**
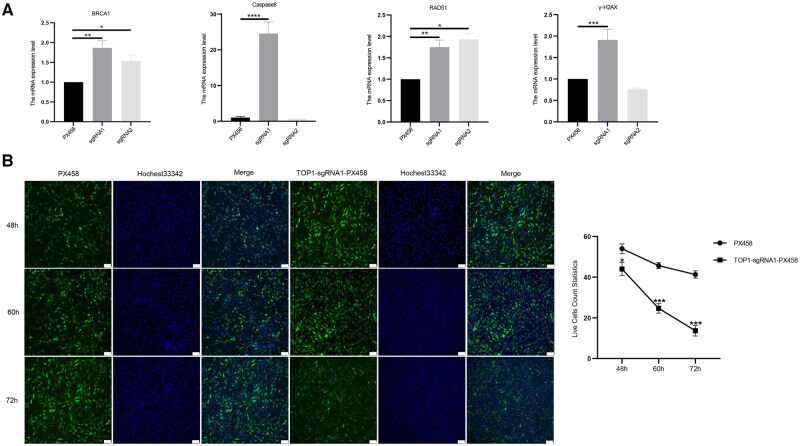
Effects of TOP1 targeting vectors on apoptosis-related gene expression and cell viability in DF-1 cells following transfection with TOP1-sgRNA-PX458 vectors. (A) Relative mRNA expression levels of apoptosis-related genes (BRCA1, Caspase8, RAD51, γ-H2AX) in DF-1 cells. sgRNA1 and sgRNA2 are samples from DF-1 cells flow cytometry sorted as positive after transfection with TOP1-sgRNA1-PX458 and TOP1-sgRNA2-PX458 vectors, respectively. Statistical significance is indicated by asterisks: *P *< 0.05 (*), *P *< 0.01 (**), *P *< 0.001 (***), *P *< 0.0001 (****). (B) Assessment of viable cell numbers in DF-1 cells transfected with TOP1-sgRNA1-PX458 vector using Hoechst 3342 staining. Left: Fluorescence images at 48, 60, and 72 h post-transfection. Green shows cell fluorescence, blue is Hoechst 3342 staining, and the merged images combine both. Right: Quantification of live cell numbers over time. PX458 is the control group, and TOP1-sgRNA1-PX458 is the experimental group. Bar = 100 μm. Statistical significance is indicated by asterisks: *P *< 0.001 (***).

To investigate whether targeted knockout of the TOP1 gene induces apoptosis in DF-1 cells, the expression of apoptosis-related genes was detected by qRT-PCR (Figure 4A) and changes in viable cell number were observed through Hoechst 33342 staining (Figure 4B). In the TOP1-sgRNA1-PX458 transfection group, the expression levels of all tested genes (Caspase 8, BRCA1, RAD51, γH2AX) were significantly higher than those in the control group (*P *< 0.01). In contrast, the expressions of BRCA1 and RAD51 were only significantly increased in the TOP1-sgRNA2-PX458 transfection groups (*P *< 0.05). These results suggest that knockout of the TOP1 gene triggers apoptosis in DF-1 cells, and the TOP1-sgRNA1-PX458 vector has the greatest impact on the expression of apoptosis-related genes in DF-1 cells after transfection. Hoechst 33342 staining results (Figure 4B) showed that the number of viable cells in the TOP1-sgRNA1-PX458 group was significantly lower than that in the PX458 control group at 48h, 60h, and 72h post-transfection (*P *< 0.01). This indicates that targeted knockout of the TOP1 gene leads to a decrease in viable cell number and the occurrence of apoptosis, with an accelerated trend in apoptosis after transfection with TOP1-sgRNA1-PX458.

**Figure 5.**
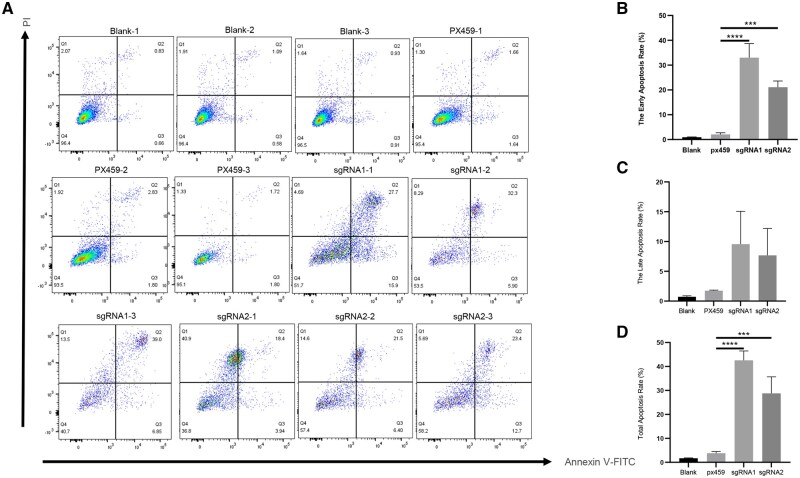
Impact of *Gg*TOP1 knockout on DF-1 cell apoptosis. (A) Flow cytometry analysis depicting the effect of TOP1-sgRNA1-PX459 and TOP1-sgRNA2-PX459 vectors on DF-1 cell apoptosis. Sub-panels show different experimental replicates for Blank, PX459 control, and experimental groups with sgRNA1 and sgRNA2. (B) Statistical analysis of early apoptosis induction in DF-1 cells following targeted *Gg*TOP1 knockout. (C) Statistical analysis of late apoptosis induction in DF-1 cells after targeted *Gg*TOP1 knockout. (D) Statistical analysis of total apoptosis induction in DF-1 cells after targeted *Gg*TOP1 knockout. In panels (B), (C), and (D), asterisks denote statistical significance: *P *< 0.001 (***).

Using an apoptosis detection kit to assess cellular apoptosis following transfection with TOP1 knockout vectors, we observed a significant increase in total apoptosis rates for TOP1-sgRNA1-PX459 and TOP1-sgRNA2-PX459 compared to the PX459 control group (*P *< 0.01), with rates of 42.55% and 28.78%, respectively (Figure 5). These results indicated that knocking out the TOP1 gene significantly induced apoptosis in DF-1 cells and further confirmed that the TOP1-sgRNA1-PX459 vector exhibits the most effective knockout efficacy.

## Discussion

TOP1 is an enzyme capable of catalyzing the coupled reaction of DNA strand cleavage and ligation, present in both eukaryotic and prokaryotic organisms. TOP1 plays crucial roles in various cellular physiological processes such as DNA replication, transcription, and repair (Rubio-Contreras and Gómez-Herreros, 2023). As a key enzyme regulating the dynamic changes in DNA spatial conformation, TOP1 influences the process of cellular apoptosis by modulating DNA topology, interacting with apoptosis-related proteins, and participating in the formation of nucleosomes (Wang et al., 2022). We found that *Gg*TOP1 shares high similarity and conservation with TOP1 from other species, leading us to infer that *Gg*TOP1 also performs similar functions in maintaining DNA stability and the normal function of genetic material.

When designing gene knockout loci, factors such as the positional relationship of exons, conserved structural regions of genes, and terminators have a significant impact on knockout efficiency. Specifically, positioning the knockout site closer to the 5′-end of the gene, within important structural domains of the functional protein, or in gene regulatory regions has always been a crucial consideration (Paolini et al., 2021). Following this principle, we successfully constructed knockout sites targeting the TOP1 gene, achieving a maximum knockout efficiency of up to 50%. Notably, the control group (PX458 vector alone) displayed markedly higher transfection efficiency than the sgRNA-expressing groups. This discrepancy may arise from this factor: the sgRNA-encoding vectors might introduce additional transcriptional or translational burdens due to the expression of both Cas9 and sgRNA, potentially affecting cellular viability or transfection competence. While the PX458 control vector lacks sgRNA, its EGFP tag still enables efficient sorting, but it does not mediate genomic cleavage, as confirmed by the absence of knockout efficiency in TA cloning and T7E1 assays. In addition, the number of Off-target matches of the sgRNA sequence should be as high as possible, and the off-target effects of the traditional CRISPR/Cas9 system cannot be ignored (Doench et al., 2016; Zhang et al., 2015) and the number of off-targets of Cas9 varies from a few to dozens, which may cause dysfunction of the body (Zhang et al., 2015). We conducted corresponding tests for predicted potential off-target sites and found no evidence of off-target effects during validation, indicating that the selected knockout sites were well-chosen. The T7E1 assay enables rapid semi-quantitative assessment of editing efficiency within one day but lacks sensitivity for SNVs and low-frequency mutations, potentially underestimating off-target effects (Sequeira et al., 2016). Although TA cloning combined with Sanger sequencing helps detect mutation types, it remains limited as a preliminary screening tool. Advanced methods like CIRCLE-seq (Lazzarotto et al., 2018) and high-throughput sequencing (Akcakaya et al., 2018; Lu et al., 2021) offer unbiased, sensitive genome-wide profiling of on- and off-target activities. Future experiments will employ deep sequencing to evaluate off-target impacts in PGC cells during sgTOP1 editing.

According to the analysis of cell apoptosis results, both sgRNAs can induce apoptosis (Figure 5A). The significant difference in the expression of apoptosis markers between sgRNA1 and sgRNA2 is likely due to the specific knockout of TOP1 functional domains and the resulting differences in DNA damage responses. sgRNA1 targets exon 4, near the N-terminus of TOP1, and its insertion/deletion mutations mainly cause frameshift mutations (such as -1 bp deletions), leading to premature termination of protein translation. This mutation completely eliminates TOP1’s ability to bind DNA, unable to repair replication-related single-strand breaks (SSBs), thereby causing accumulation of double-strand breaks (DSBs) and activating the BRCA1-dependent homologous recombination pathway and caspase-8-mediated extrinsic apoptosis pathway (Figure 4A). In contrast, sgRNA2 targets exon 13 (within the catalytic domain), and its non-frameshift mutations may retain partial DNA-binding ability but disrupt enzymatic activity. In this case, “toxic” TOP1-DNA covalent complexes are formed, stalling replication forks and inducing persistent SSBs rather than acute DSBs. At the same time, elevated levels of RAD51 and γH2AX reflect the activation of the RAD51-dependent single-strand annealing pathway and chronic replication stress, a state that tends to inhibit apoptosis and induce cell cycle arrest (Figure 4A). These findings indicate that sgRNA targeting of the initial exons of functional domains (such as sgRNA1) more effectively induces complete gene knockout and cell apoptosis, while targeting of the catalytic domain may generate hypomorphic alleles with complex off-target-like effects. The results also emphasize the importance of combining mutation type analysis (such as TA cloning) with functional assays to interpret CRISPR phenotypic results.

Currently, synthetic lethality stands as one of the methods for establishing sex control technologies across different species (Windbichler et al., 2008; Jin et al., 2013; Douglas et al., 2021). By leveraging the CRISPR/Cas9 system, sgRNA targeting critical genes for life maintenance and development can be linked to sex chromosomes along with Cas9 protein, enabling sex control in offspring through sex-linked inheritance (Windbichler et al., 2008; Jin et al., 2013; Douglas et al., 2021). In mice, by anchoring the TOP1 gene sgRNA sequence to female mice and introducing the Cas9 sequence into the Y chromosome of male mice, the combination of Cas9 (Y^Cas9^) and sgRNA (X^TOP1-sgRNA^) were utilized knock out the TOP1 gene upon hybridization, resulting in early embryonic lethality in males (X^TOP1-sgRNA^ Y^Cas9^) and the production of only female mice (Douglas et al., 2021). Utilizing the CRISPR/Cas9 system to create a Y-chromosome-linked Cas9, single-sex selection was conducted, and gene drive in *Drosophila*, and the researchers successfully identified the sex development of fertilized eggs through molecular biological techniques, laying the foundation for the application of the Y-chromosome-linked Cas9 system (Chen et al., 2022). Our results also demonstrated that the *Gg*TOP1 gene could induce apoptosis in DF-1 cells. Therefore, our next step will be to establish a synthetic lethal model in chicken using TOP1 (Supplementary Figure S4), design a two-component synthetic lethal system using CRISPR–Cas9, and utilize Cas9 specifically expressed on sex chromosomes to pave the way for controlling the birth of a single-sex offspring. Such concepts and methods provide new possibilities for sex control in poultry.

## Conclusions

The present study, through the construction and validation of an efficient *TOP1* gene knockout vector, for the first time unveils the pivotal role of the chicken TOP1 gene in apoptosis of DF-1 cells. The results demonstrate that TOP1 gene knockout significantly promotes cell apoptosis, confirming its crucial function in maintaining cell survival. This finding provides a new perspective for the investigation of the *TOP1* gene function and the mechanisms underlying cell fate regulation. Furthermore, it lays a foundation for future applications in the production of monosexual chickens, gene therapy, and cellular engineering.

## Supplementary Material

skaf315_Supplementary_Data

## Data Availability

None of the data were deposited in an official repository. The datasets generated and analyzed during the current study are available from the corresponding author upon request.

## Abbreviations:

TOP1: topoisomerase I
sgRNA: single-guide RNA
CRISPR-Cas9: clustered regularly interspaced short palindromic repeats/CRISPR-associated protein 9
CDS: coding sequence
qRT-PCR: quantitative real-time PCR
T7E1: T7 endonuclease I
