# Supplementary material for: CRISPR/Cas9-mediated TOP1 knockout in chicken DF-1 cells reveals its critical role in apoptosis regulation and genomic stability
Source: J Anim Sci. 2025 Sep 15;104:skaf315. doi: 10.1093/jas/skaf315 (PMC12863936; doi:10.1093/jas/skaf315)
Supplement: skaf315_Supplementary_Data [file skaf315_supplementary_data.zip › 04-Dec-2025_051351_Supplementary_Figure_Legends.docx]

# Supplementary Figure Legends

Figure S1. Molecular features of *Gg*TOP1 and *Ms*TOP1. (A) Amino acid sequence alignment of *Gg*TOP1 (*Gallus gallus*, NP_990441.1) with TOP1 from other species: *Homo sapiens* (NP_003277.1), *Mus musculus* (NP_033434.2), *Bos taurus* (NP_001193416.1), *Sus scrofa* (XP_020934054.1), *Taeniopygia guttata* (XP_002190040.2), *Anas platyrhynchos* (XP_027328739.1), *Danio rerio* (NP_001037789.1), *Drosophila melanogaster* (NP_727841.1), *Xenopus laevis* (NP_001084031.1), *Lethenteron reissneri* (XP_061421745.1), *Magallana gigas* (XP_011445668.3), *Caenorhabditis elegans* (NP_001379240.1). (B)Phylogenetic tree analysis depicting the evolutionary relationship of *Gg*TOP1 with TOP1 from other species. The tree was constructed to illustrate the genetic relatedness among these sequences. (C)Predicted structural domains of *Gg*TOP1. The diagram shows the different functional regions within the *Gg*TOP1 protein. (D) Tertiary structure prediction of *Gg*TOP1 and *Ms*TOP1 (*Mus musculus* TOP1) using SWISS - MODEL. The α - helices are colored red, coils are gray, β - pleated sheets are blue, and turns are green, providing a visual representation of the proteins’3D conformations.

Figure S2. Prediction of off - target sites for the three designed sgRNAs. (A) Prediction of off-target sites for TOP1-sgRNA1. The table lists coordinates, strand information, Mismatch (MM) details, target sequences, Protospacer Adjacent Motif (PAM) sequences, distance values, gene names, and gene IDs. The yellow - highlighted sections denote specific off - target sites chosen for in - depth investigation in this study. (B) Prediction of off-target sites for TOP1-sgRNA2. Similar to (A), this table presents relevant details for potential off - target sites, with the yellow highlighted areas indicating the specific off-target sites selected for detailed analysis in the research.

Figure S3. Detection of off - target probabilities for TOP1-sgRNA2 at predicted sites. (A-C) For genes with IDs ENSGALG00000007547, ENSGALG00000049997, and ENSGALG00000009168 respectively. Left: T7E1 digestion gel (M=marker). Right: TA cloning sequencing of target sequences (blue boxes highlighted target sequences)) to assess off-target effects.

Figure S4: This diagram illustrates the experimental process for gene - editing in chickens related to the TOP1 gene. Primordial germ cells (PGCs) are introduced with either Z^TOP1−sgRNA^Z^TOP1−sgRNA^ or Z^Cas9^W. F1 generation chimeric chickens are generated, followed by the production of F2 gene - edited chickens through test cross and sequencing screening. The red "×" indicates an outcome that is not viable or is being excluded in the process. This figure also provides a preview of how the TOP1 gene will be harnessed in future work, based on the principle of synthetic lethality, to achieve control over poultry sex ratio.
