# Supplementary material for: CRISPR/Cas9-mediated TOP1 knockout in chicken DF-1 cells reveals its critical role in apoptosis regulation and genomic stability
Source: J Anim Sci. 2025 Sep 15;104:skaf315. doi: 10.1093/jas/skaf315 (PMC12863936; doi:10.1093/jas/skaf315)
Supplement: skaf315_Supplementary_Data [file skaf315_supplementary_data.zip › Supplement_Data.zip!/Supplement Tables-20250903.docx]

**Table S1.** Targeted Knockout of Enzyme Digestion base sequences.

| Gene | The sequence of Enzyme Digestion base sequences（5’→3’） |
| --- | --- |
| chicken-PX458-TOP1-1 | F: CACCGCAAAGACAAGGAGAAGAGAAAGG |
|  | R: AAACCCTTTCTCTTCTCCTTGTCTTTGC |
| chicken-PX458-TOP1-2 | F: CACCGGGAATATGGTTACTGTGTGATGG |
|  | R: AAACCCATCACACAGTAACCATATTCCC |
| chicken-PX458-TOP1-3 | F: CACCGGGAGGTCCGGCATGATAACAAGG |
|  | R: AAACCCTTGTTATCATGCCGGACCTCCC |

**Table S2.** The sequence of qRT-PCR primers.

| Gene | The sequence of qRT-PCR primers（5’→3’） |
| --- | --- |
| β-actin | F: CAGCCATCTTTCTTGGGTAT |
|  | R: CTGTGATCTCCTTCTGCATCC |
| caspase8 | F: GCCTTCTTCCAAGCATTAC |
|  | R: AGATACCTGAACGGAGACA |
| JUN | F: GCGGCTATGGATATAATAATC |
|  | R: TGCTGGACTGGATGATGA |
| BRCA1 | F: TTGTGGTGCCTCTGTAGT |
|  | R: CGCTGGATTGCTTCATAAC |
| γ-H2AX | F: CTAGCCAGATCGGACTCA |
|  | R: GTGCGACCTATCATCATCT |

**Table S3.** TA clone/T7E1 enzyme digestion amplification primer sequences.

| Gene | The sequence of TA clone/T7E1 enzyme digestion amplification primers（5’→3’） |
| --- | --- |
| TA/T7E1-sgRNA1 | F: ATCGTAACTTCTGGCTCTG |
|  | R: CTGCTGTGGAACTGTCTC |
| TA/T7E1-sgRNA2 | F: GATGTTCCTCTGCTTGCT |
|  | R: GGAAGCACAAGTAGAGGAA |

**Table S4.** Enzyme digestion system and grouping (10.5μL).

| Group | Test group (Lane 1) | Hybrid group (Lane 2) | Control group (Lane 3) | Blank group (Lane 4) |
| --- | --- | --- | --- | --- |
| Mutant DNA fragment (ng) | 150ng | 75ng | 0 | 150ng |
| Wild-type DNA fragment (ng) | 0 | 75ng | 150ng | 0 |
| 10 × Buffer | 1.1μL | 1.1μL | 1.1μL | 1.1μL |
| RNase-free ddH_2_O | Up to 10.5μL | | | |

**Table S5. The sequence of Off target Site Primer.**

| Gene | The sequence of Off target Site Primer（5’→3’） |
| --- | --- |
| sgRNA1- ENSGALG00000050841 | F: GTTGTGTGGGTAGAAGATAGA |
|  | R: CACAAGTGAGAATCATTGGA |
| sgRNA1- ENSGALG00000012683 | F: TTAACGAAGCCTGTTCTGT |
|  | R: TAATCACCTGCCGTTGAAT |
| sgRNA1- ENSGALG00000012319 | F: CTGAAGGACGCTATACTGAT |
|  | R: GTAATGACTGGCAACACTC |
| sgRNA2- ENSGALG00000007547 | F: ATGACCTCTGGATTAACTGT |
|  | R: CACTGACTTGAAGACTCCT |
| sgRNA2- ENSGALG00000049997 | F: GCAGAGTGGTGTGACTTC |
|  | R: GATTCCATCTCTACGCCTAC |
| sgRNA2- ENSGALG00000009168 | F: AACTGCTTGAGTATCCTGAA |
|  | R: TGTGCTCTGTGGTTATCTC |
